# Comparable contributions of structural-functional constraints and expression level to the rate of protein sequence evolution

**DOI:** 10.1186/1745-6150-3-40

**Published:** 2008-10-07

**Authors:** Maxim Y Wolf, Yuri I Wolf, Eugene V Koonin

**Affiliations:** 1 National Center for Biotechnology Information, National Library of Medicine, National Institutes of Health, Bethesda, MD 20894, USA

## Abstract

**Background:**

Proteins show a broad range of evolutionary rates. Understanding the factors that are responsible for the characteristic rate of evolution of a given protein arguably is one of the major goals of evolutionary biology. A long-standing general assumption used to be that the evolution rate is, primarily, determined by the specific functional constraints that affect the given protein. These constrains were traditionally thought to depend both on the specific features of the protein's structure and its biological role. The advent of systems biology brought about new types of data, such as expression level and protein-protein interactions, and unexpectedly, a variety of correlations between protein evolution rate and these variables have been observed. The strongest connections by far were repeatedly seen between protein sequence evolution rate and the expression level of the respective gene. It has been hypothesized that this link is due to the selection for the robustness of the protein structure to mistranslation-induced misfolding that is particularly important for highly expressed proteins and is the dominant determinant of the sequence evolution rate.

**Results:**

This work is an attempt to assess the relative contributions of protein domain structure and function, on the one hand, and expression level on the other hand, to the rate of sequence evolution. To this end, we performed a genome-wide analysis of the effect of the fusion of a pair of domains in multidomain proteins on the difference in the domain-specific evolutionary rates. The mistranslation-induced misfolding hypothesis would predict that, within multidomain proteins, fused domains, on average, should evolve at substantially closer rates than the same domains in different proteins because, within a mutlidomain protein, all domains are translated at the same rate. We performed a comprehensive comparison of the evolutionary rates of mammalian and plant protein domains that are either joined in multidomain proteins or contained in distinct proteins. Substantial homogenization of evolutionary rates in multidomain proteins was, indeed, observed in both animals and plants, although highly significant differences between domain-specific rates remained. The contributions of the translation rate, as determined by the effect of the fusion of a pair of domains within a multidomain protein, and intrinsic, domain-specific structural-functional constraints appear to be comparable in magnitude.

**Conclusion:**

Fusion of domains in a multidomain protein results in substantial homogenization of the domain-specific evolutionary rates but significant differences between domain-specific evolution rates remain. Thus, the rate of translation and intrinsic structural-functional constraints both exert sizable and comparable effects on sequence evolution.

**Reviewers:**

This article was reviewed by Sergei Maslov, Dennis Vitkup, Claus Wilke (nominated by Orly Alter), and Allan Drummond (nominated by Joel Bader). For the full reviews, please go to the Reviewers' Reports section.

## Background

The first grand generalization of molecular evolution is that proteins evolve at widely different rates but each particular protein has a characteristic rate that remains relatively constant over long evolutionary spans [1]. In other words, there seems to exist a molecular clock that ticks at widely different paces for different protein-coding genes. What determines this characteristic rate is, arguably, one of the central questions of evolutionary biology. To our knowledge, the first explicit hypothesis on the interplay of factors that determine the rate of a protein's evolution belongs to Wilson et al. who proposed, in their classic review on molecular evolution, that the sequence evolution rate should be a function of, firstly, the intrinsic functional constraints that affect the given protein and, secondly, on the biological function of the protein in the organism: *R*_*i *_= *f*(*P*_*i*_)*f*(*Q*_*i*_) where *R*_*i *_is the sequence evolution rate, *f*(*P*_*i*_) is the functional-constraint factor, and *f*(*Q*_*j*_) is the dispensability factor [2]. Testing this hypothesis at the time was hardly feasible, so given that the functions and structures of proteins are, indeed, widely different and so are the rates of sequence evolution, it was (more or less) tacitly assumed that the first term in Wilson's equation was the decisive one.

Things changed with the advent of functional genomics and systems biology in the beginning of the 21^st ^century when it became possible to measure the correlations between many "genomic" variables [3-7]. Quite surprisingly, it turned out that there was little if any correlation between the essentiality of genes for the reproduction of organisms and their rates of evolution: at best, non-essential genes evolve slightly faster than essential genes [8-13]. Equally unexpectedly, highly significant negative correlation has been shown to exist between a gene's expression level and its evolutionary rate, that is, highly expressed genes evolve significantly slower than lowly expressed ones [11,14-16]. Many other correlations between genomic variables have been examined including but not limited to the number of protein-protein and genetic interactions, position in various kinds of networks, and the propensity to be lost during evolution [11,17-22]. Generally, the evolutionary variables, namely, the sequence evolution rate and the propensity for gene loss, are positively correlated with each other and negatively correlated with "phenomic" variables such as expression level, number of interactions and others [4,23].

Most of the observed correlations between genomic variables are relatively weak. The link between expression level and sequence evolution rate appears to be by far the strongest and most consistent across a range of diverse organisms, a finding that led to the striking hypothesis that expression level or, more precisely, the rate of translational events is indeed the dominant determinant of the sequence evolution rate [15,24,25]. It has been further hypothesized that the underlying cause of the covariation between the sequence evolution rate and expression level is the selection for robustness to protein misfolding that is increasingly important for highly expressed proteins owing to the toxic effects of misfolded proteins [25,26]. Misfolded forms of highly expressed proteins are thought to be strongly deleterious for a cell, so there could be a strong selection to avoid their accumulation. Recent detailed computer simulations of protein evolution suggest that the toxic effect of protein misfolding, indeed, could suffice to explain the observed covariation of expression level and sequence evolution rate [25].

The hypothesis on the crucial role of the selection for robustness to misfolding, dependent on the translation rate, in protein sequence evolution is a drastic departure from the more traditional thinking that links the evolution rate, primarily, to intrinsic structural-functional constraints that are thought to substantially differ for different proteins [2,27]. We were interested in directly assessing the relative contributions of effects mediated by the translation rate and intrinsic structural-functional constraints to the evolution rates of protein sequences. The results of the analysis described here indicate that both contributions are substantial and comparable in magnitude.

## Results

### Rationale and Approach

This analysis relies on the high abundance and diversity of multidomain proteins in eukaryotes [28,29]. The simple underlying idea is that different domains of the same protein are translated at the exact same rate. Accordingly, under the mistranslation-induced misfolding (hereinafter MIM) hypothesis, distinct domains within the same multidomain protein, on average, would be expected to evolve at substantially closer rates than the same domains when contained in different proteins. In the extreme, that is, under the obviously over-simplifying assumption that the intrinsic structural-functional constraints that affect different domains are the same, the constituent domains of any multidomain protein would evolve at the same rate (within sampling error). Conversely, the remaining difference in the evolution rates of domains in multidomain proteins is attributable to the differences in the intrinsic constraints. So, at least, in principle, by measuring the extent of "homogenization" of evolutionary rates of domains in multidomain proteins, it should be possible to disentangle and compare the contributions of translation rate-mediated factors and translation-independent, intrinsic ones.

**Figure 1:**
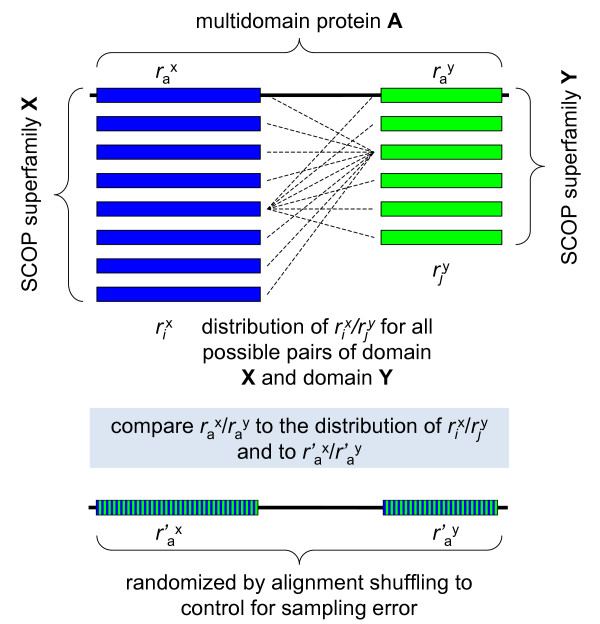
**Scheme of the comparison of evolution rates of domains located in multidomain proteins and in separate proteins**. *r*_*i*_^x ^– evolutionary rates of all instantiations of domain X in human (*Arabidopsis*) proteins; *r*_*j*_^y ^– evolutionary rates of all instantiations of domain Y in human (*Arabidopsis*) proteins; *r*_a_^x ^– evolutionary rate of domain X in multidomain protein A; *r*_a_^y ^– evolutionary rate of domain Y in multidomain protein A; *r*'_a_^x ^– evolutionary rate of the randomized sequence segment within the boundaries of domain X in multidomain protein A; *r*'_a_^y ^– evolutionary rate of the randomized sequence segment within the boundaries of domain Y in multidomain protein A.

The approach is schematically illustrated in Figure 1. In addition to the potential for directly assessing the contributions of translation rate and domain identity (structural-functional constraints), this approach has the major advantage of not relying on experimental gene expression data that are, inevitably, noisy. Furthermore, the interpretation of experimental data on gene expression is ambiguous because the quantity that is actually measured in most experiments is the transcript level rather than the number of translation events per se, which is the decisive factor under the MIM hypothesis [25,26].

The only substantial caveat we are aware of is the possibility that, because of alternative splicing, different domains of some multidomain proteins are actually expressed at different rates. However, it seems unlikely that alternative splicing would have a major effect on the results considering that there is limited correspondence at best between alternative splice form structure and domain architectures of proteins [30]. Furthermore, as described below, we performed the comparisons of fused and separated domains in both mammals where alternative splicing is most abundant and in plants where it is thought to be much less common [31].

### Comparison of the evolutionary rates of fused and separated protein domains

In order to implement the scheme shown in Figure 1, we mapped structurally distinct domains from the SCOP/ASTRAL database onto alignments of orthologous proteins from human and mouse, and Arabidopsis and cottonwood. Evolutionary rates were calculated for the complete sets of domains, domains fused within multidomain proteins, and domains contained in different proteins (see Materials and Methods for details). All the analyses described below were performed on protein superfamilies, the intermediate level of the structural classification of proteins implemented in the SCOP database [32]; however, very similar results were obtained with the higher (fold) and lower (family) levels of proteins classification (data not shown).

**Figure 2:**
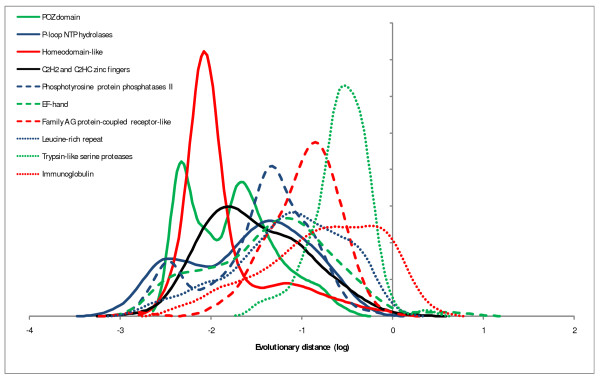
**Evolution rate distributions of highly abundant domains**. The rates were estimated from comparisons of human-mouse orthologous protein sequences. X-axis: log_10 _of domain evolution rate, Y-axis: probability density function..

**Figure 3:**
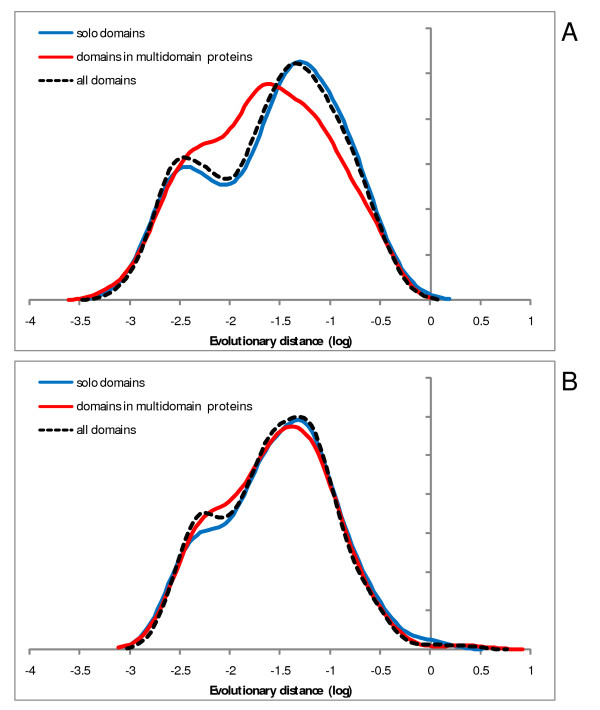
**Evolutionary rate distributions for two superfamilies of highly abundant domains: comparison of fused and solo domains**. A – P-loop-containing NTP hydrolases; B – PH-like domains. The rates were estimated from comparisons of human-mouse orthologous protein sequences. X-axis: log_10 _of domain evolution rate, Y-axis: probability density function.

Examination of the distributions of evolutionary rates among different domains is interesting in itself: mean evolutionary rates of abundant domains show a span of almost 3 orders of magnitude (Figure 2), in a qualitative agreement with previous observations made by comparison within protein families [33] or functional categories of genes [34]. The distributions typically are broad but follow a general bell-shaped form (some are bimodal), in support of the notion of an intrinsic rate of domain evolution for which the mean (or median) of the respective distribution can be taken as a reasonable proxy. Domains present within multidomain proteins do not show systematic differences in evolutionary rates compared to solo domains as illustrated by two examples in Figure 3.

**Figure 4:**
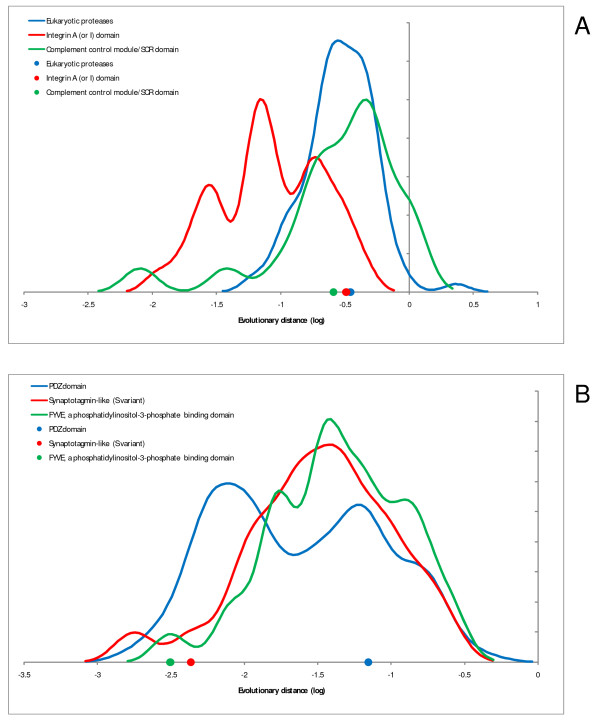
**Examples of domain evolutionary rates in multidomain proteins**. A – complement component 2 precursor (C2; NP_000054). B – protein regulating synaptic membrane exocytosis 2 (RIMS2; NP_001093587). The curves indicate the rate distributions for the constituent domains of multidomain proteins (as in Figure 2 dots indicate the rates for the corresponding domains (color-coded) in the given protein.

**Figure 5:**
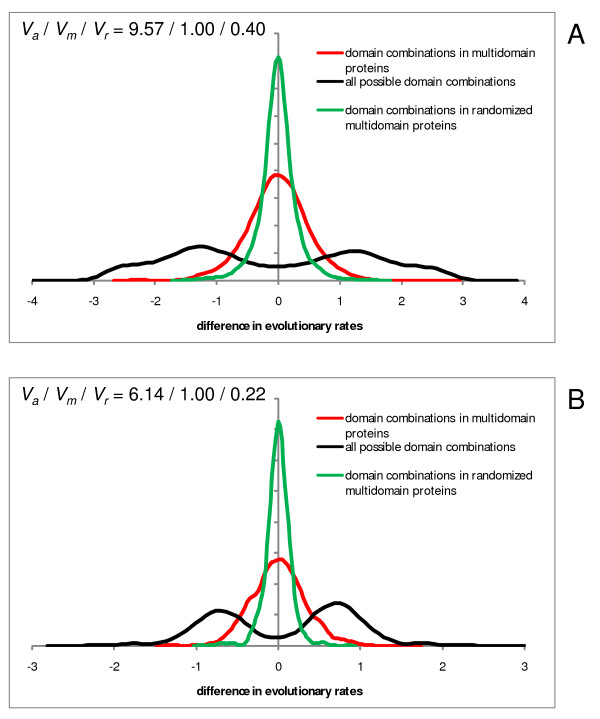
**Differences of evolution rates for domains contained in the same multidomain protein and in different proteins, compared to randomized "domain" sequences from multidomain proteins**. X-axis: log_10 _of the ratio of evolution rates of a pair of domains. Y-axis: probability density function. Red, domain pairs within multidomain proteins; black, all possible pairwise combinations of the same domains (from both multidomain proteins and proteins in which the respective domains occur separately); green, randomized domains within multidomain proteins (control for sampling error). The randomized domains were obtained by randomly shuffling the columns in a multidomain protein alignment, and evolution rates were calculated for regions within the original domain boundaries. *V_a_*, *V_m_*, and *V_r_* are the normalized variances of the distributions for all domain pairs, domain pairs within multidomain proteins, and randomized domains, respectively. A – Human proteins: *V*_*a*_/*V*_*m*_/*V*_*r *_= 9.57/1.00/0.40. B – *Arabidopsis *proteins: *V*_*a*_/*V*_*m*_/*V*_*r *_= 6.14/1.00/0.22.

We then addressed the issue of homogenization of the rates of evolution of domains that is predicted by the MIM hypothesis to result from the fusion of domains within a multidomain protein. Figure 4 shows anecdotal evidence for two proteins, each consisting of 3 distinct domains. For one of these proteins, homogenization is obvious (Figure 4A) whereas the other one shows no obvious sign of homogenization (Figure 4B). These examples are characteristic of the diversity of the evolutionary regimes of domains, so that homogenization is seen in many but by no means all multidomain proteins, and some actually display the opposite trend (Additional Files 1 and 2, and see below). This striking variability notwithstanding, the results of the analysis of the complete sets of domains unequivocally reveal substantial homogenization as illustrated by the comparison of the probability density functions for the difference (ratio) of the evolutionary rates for all domain combinations and for domain pairs fused within multidomain proteins. The difference in evolutionary rates between a pair of domains within a multidomain protein tends to be substantially less than the difference between rates for the same pair of domains found in different proteins in both human (Figure 5A) and *Arabidopsis *(Figure 5B).

However, homogenization is far from being complete as shown by comparing the distributions of rate differences between domains in multidomain proteins to control distributions obtained with sequence segments within the original domain boundaries in randomized alignments of multidomain proteins. The variance of the distribution of the rate differences between the domains of multidomain proteins was ~2.5 times greater than the variance for the control distribution for human proteins (Figure 5A) and ~4.4 times greater in the case of *Arabidopsis *(Figure 5B). Taken together, these findings indicate that fusion of domains in multidomain proteins leads to substantial homogenization of their evolutionary rates although highly significant rate differences between the domains remain.

**Figure 6:**
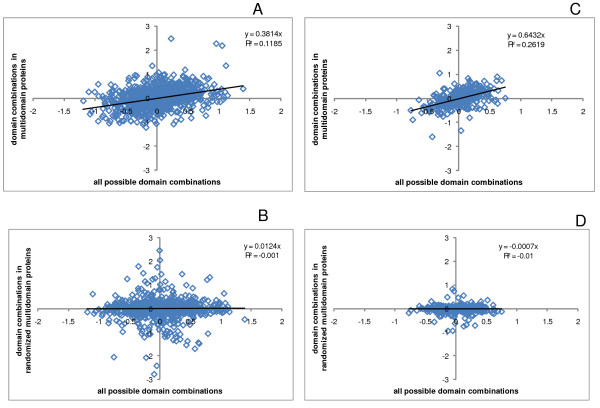
**Correlations between the evolutionary rate differences of domains within multidomain proteins and the same domain pairs from separate proteins**. Each data point corresponds to a pair of SCOP superfamilies (S^1^, S^2^) that is observed at least once in the same multidomain protein in a genome. X-axis: mean of log_10 _of the ratios of evolution rates of all combination of domains (D_*i*_^1^, D_*j*_^2^) where D_*i*_^1 ^∈ S^1 ^and D_*j*_^2 ^∈ S^2^. Y-axis: mean of log_10 _of the ratios of evolution rates of all combination of domains (D_*i*_^1^, D_*j*_^2^) where D_*i*_^1 ^∈ S^1^, D_*j*_^2 ^∈ S^2 ^and (D_*i*_^1^, D_*j*_^2^) belong to the same multidomain protein. A – Human proteins. B – Randomized human protein sequences. C – *Arabidopsis *proteins. D – Randomized *Arabidopsis *protein sequences.

We then examined the correlations between the mean rate differences of domain pairs that are conjoined in multidomain proteins and the same domain pairs found in different proteins. If fusion within a multidomain proteins, on average, has no effect on the evolutionary rates of the domains involved, the geometric mean of the ratio of the rates for the given pair of domains should be equal to that for all combinations of these domains (up to the sampling error), so the slope of the regression line is expected to be equal to 1. Conversely, if the evolution rates of the constituent domains in multidomain proteins are completely homogenized, all rate differences for domain pairs in multidomain proteins should be close to 0 (again, subject to a sampling error), and the slope of the regression line would be equal to 0 as well. The results show that both in human (Figure 6A) and in *Arabidopsis *(Figure 6C), there is a limited but statistically highly significant, positive correlation between the rate differences of domains in the two classes of domain pairs. This correlation was in a sharp contrast with the results of similar comparisons that were performed with sequences of multidomain proteins that were randomized over the entire lengths and the rates of evolution were then compared for regions within the original domain boundaries (compare Figure 6A with 6B, and Figure 6C with 6D). The slope of the linear trendline in the log-log scale was ~0.38 for the 963 domain pairs in human genome and ~0.64 for the 355 domain pairs in *Arabidopsis *genome. For instance, if the mean evolution rates of two domains in human proteins differ by a factor of 2, then an ~1.3 fold difference in rates can be expected when these domains are fused within a single multidomain protein; similarly, in the case of *Arabidopsis*, an ~1.6 fold difference can be expected. These results indicate that the contributions of translation-rate related factors and intrinsic structural-functional constraints to the rate of protein sequence evolution are comparable.

## Discussion

The comparative analysis of the differences between domain evolution rates within multidomain proteins and in separate proteins reveals comparable contributions of factors that we consider to be translation rate-related (and hence uniformly affecting all domains of a multidomain protein) and intrinsic factors that differ between domains, regardless of whether or not they belong to the same multidomain protein. Continuing the line of thought under which selection for tolerance to amino acid misincorporation (caused by mistranslation, transcription error or mutation), or misfolding robustness, is the dominant factor of evolution [24,25], it seems possible to interpret these results in terms of a generalized MIM hypothesis. Thus, the rate of protein sequence evolution would be considered to depend on two factors:

(i) Intrinsic misfolding robustness that depends on the structure of the given domain, and in particular, on its characteristic stability and designability [35]. It has been shown that proteins (or domains within multidomain proteins) with a greater amino acid residue contact density (more designable ones.) evolve faster than less designable proteins (domains) although the effect was relatively small and differed greatly among organisms [36,37]. Misfolding of a protein molecule is assumed to incur a specific fitness cost which might be unrelated to the protein's function, such as through a cytotoxic effect of the misfolded protein [25].

(ii) Translation rate that serves as an amplifier of the fitness cost of misfolding (roughly, the total cost is at least proportional to the number of translation events, considering that the error rate of translation is orders of magnitude greater than those of replication and transcription [38]) and, accordingly, of the strength of selection for the robustness to amino acid misincorporation.

The analysis of evolutionary rates of individual domain in multidomain proteins allows one to disentangle the two factors by removing the effect of translation rate (the amplifier). The results show that the remaining difference in evolutionary rates of domains is much less than the total difference, that is, the amplifying effect of translation is substantial.

The MIM hypothesis is a highly attractive concept not only because it introduces a single, dominant determinant of protein evolutionary rate but also because the key role of misfolding robustness is compatible with fundamental biological features of all cells. Indeed, all cells encode numerous chaperones that prevent misfolding and enormously elaborate molecular machines, such as proteasomes, that to a large extent are dedicated to the selective degradation of misfolded proteins [39].

Nevertheless, for a more complete explanation of the observed variation of protein (domain) evolution rates, it might be desirable to take into account other extrinsic variables, in addition to the expression level, such as the number of physical and genetic interactions, functional importance (fitness cost of knockout), and perhaps, others. An example of such a pluralistic explanatory framework is the concept of a gene's "status" according to which different extrinsic variables independently but concordantly contribute to sequence evolution [23].

We showed here that the contributions of intrinsic and extrinsic factors to the rate of protein sequence evolution are of comparable magnitude. Definitive, concrete interpretation of these terms and decomposition of each of them into more specific contributions are crucial goals for further experimental, computational and theoretical studies that ultimately might allow us to reach an adequate understanding of protein evolution.

## Materials and methods

A non-redundant set of human protein sequences (one per locus) and a set of *Arabidopsis *protein sequences (both from NCBI RefSeq release 29) were used as queries in a BLASTP search [40] with the e-value threshold of 0.0001 against a database consisting of ASTRAL 1.73 domain sequences [41]. A non-overlapping set of best ASTRAL hits was used to map SCOP 1.73 [42] domains to the human and Arabidopsis proteins. "Double-solo" domains, that is, domains belonging to a single-domain protein that was the only representative of the respective SCOP family, were discarded. This procedure yielded 9786 domains from 1111 different SCOP families that mapped to 7404 human proteins and 5908 domains belonging to 1035 different SCOP families that mapped to 5118 *Arabidopsis *proteins.

In parallel, a set of orthologs for human and *Arabidopsis *proteins was obtained using the bi-directional best hit scheme [43] against a set of mouse (NCBI RefSeq release 29) and cottonwood (DOE Joint Genome Institute *Populus *genome release 1.1) proteins, respectively. This procedure yielded 16,603 human-mouse and 15,286 *Arabidopsis*-poplar orthologous protein pairs. Pairwise alignments of the orthologous protein sequences were generated using the MUSCLE program [44].

Segments of human-mouse (*Arabidopsis*-cottonwood) ortholog alignments that corresponded to the SCOP domains were extracted, and the evolutionary distances between human and mouse (*Arabidopsis *and cottonwood) domains were calculated using the PROTDIST program of the PHYLIP package (JTT evolutionary model; gamma-distributed site rates with shape parameter 1.0) [45]. To control for sampling error, full-length alignments were randomized by permuting the alignment columns after which the domains were extracted using the same coordinates as in the original alignment, and the distances were calculated as indicated above.

Smoothing of the individual data points to produce the distribution curves shown in Figures 2, 3, 4, 5 was performed using the Gaussian-kernel method [46].

## Competing interests

The authors declare that they have no competing interests.

## Authors' contributions

MYW and YIW performed the data collection and analysis. YIW and EVK designed the analysis and interpreted the results. EVK conceived of the study and wrote the manuscript. All authors read and approved the final version.

## Reviewers' comments

### Reviewer's report 1: Sergei Maslov, Brookhaven National Laboratory

The manuscript reports a simple yet conclusive test of whether the variability of rates of protein evolution can be explained exclusively by the difference in their expression levels. The unequivocal answer is: "No".

This conclusion comes from comparison of the evolutionary rates of different domains within the same multi-domain protein, which obviously have identical expression levels. Authors find that while the intraprotein domain-to-domain variability of evolutionary rates (black curve in Figure 5) is dramatically reduced compared to that of the same domains in different proteins (red curve in Figure 5), it still remains considerably broader than in the null-model (green curve in Figure 5). In this null-model authors simulate the variability due to the sampling error by first reshuffling each of the alignments over the whole aligned region encompassing both domains and then recalculating the variability of the resulting homogenized domains. The fact that the null-model variability is smaller than that of the actual alignment indicates that some systematic difference in evolutionary rates of different domains still remains even when their expression levels are identical. This residual variability is tentatively attributed to domain-specific structural-functional constraints.

The null-model itself is very important and (in my view) it is not adequately explained in the text of the manuscript. Indeed, the fact that the domain-to-domain variability of evolutionary rates is considerably broader than in the null-model is one of the central observations of the manuscript. Even though the Methods section contains a more detailed description of the null-model, I feel that authors should outline it in a few sentences right where it is first mentioned in the manuscript. This description should make it clear that the alignments are reshuffled over the entire aligned region encompassing all of the domains. Such reshuffling effectively homogenizes the evolutionary rates of individual domains.

**Authors' response: ***such a description was added and, hopefully, clarifies the presentation of the results*.

My main comment on the manuscript is regarding the claim made in the title, the abstract, and many times in the main text. Authors claim that the relative contributions of the expression level and the domain-specific structural-functional constraints to variability of evolutionary rates are *closely comparable*. If closely comparable means that, when considered separately, they reduce the variability by approximately equal factors, then it is not at all obvious from the manuscript. From Figure 5 one gets the impression that the reduction in variability when the expression level differences are taken into account is much stronger than the residual variability due to structural-functional constraints. Could authors perhaps make a more quantitative comparison of relative contributions of these two factors?

**Authors' response: ***Actually, the comparison of the contributions of translation rate and structural functional constraints is quantitative as illustrated in Figure *6. *To quote: "For instance, if the mean evolution rates of two domains in human proteins differ by a factor of 2, then an ~1.3 fold difference in rates can be expected when these domains are fused within a single multidomain protein; similarly, in the case of Arabidopsis, an ~1.6 fold difference can be expected." We believe that this comparison justifies the conclusion that these contributions are indeed comparable. However, we removed the claim that they are "closely" comparable. Furthermore, in the revised manuscript, we provide a quantitative comparison in Figure *5*as well, in terms of the normalized variance of the distributions of evolutionary rate ratios. The results obtained with these two approaches are compatible, at least, qualitatively*.

My second comment or rather a question to the authors: does taking the expression level into account explains all of the variability of *silent* substitution rates? That is what one expects if all of the residual variability visible in Figure 5 is due to structural-functional constraints.

**Authors' response: ***This certainly deserves to be tested. However, the prediction is not as clear-cut as it might immediately seem because the selection pressure that affects silent positions (that is, codon usage) could actually depend on the intrinsic tolerance of domain to amino acid misincorporation. So for the moment we decided that this analysis was not an integral part of the study*.

### Reviewer's report 2:Dennis Vitkup, Columbia University

The paper by Maxim Wolf *et al*. investigates one of the main puzzles of molecular evolution – the existence of the protein-specific molecular clock. While the rate of evolution for different proteins (genes) varies by several orders of magnitude, it seems to be roughly constant for orthologous protein in different species. This observation, first made in the sixties, begs for an explanation. What factors set the rate of accepted mutations for each protein? I think it was first demonstrated by Laurence Hurst et al. in 2001 that a major determinant of the clock rate is gene expression. It accounts for about 50% of the rate variance among different proteins. Other functional determinants (protein length, number of protein-protein interactions, metabolic flux, etc) were shown by other studies to account for smaller fractions of the rate variance (usually between 5%–10%).

Recently, Drummond *et al*. (2005 PNAS, 2008 Cell) proposed an interesting hypothesis that the clock rate depends strongly on gene expression primarily due to the protein misfolding. Specifically, the evolution is significantly constrained by the optimality of codons used in each protein. Non-optimized codons will generate a significant fraction of toxic (due to protein aggregation) misfolded proteins. The amount of misfolded proteins is proportional to the number of translational events and consequently depends on gene expression.

One comment I have about the paper is that it primarily tests the independent contribution of the gene expression versus other structural-functional constraints. It does not test the validity of the misfolding hypothesis that expression affects the evolutionary rate *primarily *through misfolding. The authors need to make this very clear. There may be many other explanations for the observed expression-rate correlation, for example, energy conservation. As far as I know, currently there are no experimental confirmations of the proposed misfolding theory.

I think the method used to investigate the homogenization of rates is quite elegant. I think that in addition to the fact that, as authors state, "the interpretation of experimental data on gene expression is ambiguous because the quantity that is measured in most experiments is the transcript level rather than the number of translation events", the analysis of the multi-domain proteins escapes the difficulty that expression of any protein is very heterogeneous in many different conditions. There is no single expression level for a protein or a domain. Analysis of domain fused into a single ORF insures that they are present in exactly the same amount (or require similar number of translation events) under all possible environmental, developmental, and regulatory conditions. Maybe authors need to add a comment about this.

I think another important caveat is that the fusion of domains into a single protein usually ensures, at least, partial homogenization of protein function. This is important as authors do not check that the fused domains have the same molecular functions. For example, in metabolic network fused domain are almost always direct neighbors in a metabolic pathway. In contrast, single domain with the same structure (from the same SCOP family) may be responsible for very different metabolic functions. The same is certainly true for other (non-metabolic) proteins. Consequently, the homogenization of the evolutionary rate specifically due to expression will be estimated from above. The real expression-based homogenization will be lower.

It is interesting to note that in the Figure 2 the majority of protein domains demonstrate multimodal behavior. I think all distributions are clearly multi-modal, although with different peak weights. It may be more logical to use the lower SCOP hierarchy for the analysis in which most distributions are single peaked. But the authors state that similar results were obtained with a lower level of the SCOP classification.

In future studies it may be also interesting to investigate the rate homogenization due to tight (non-transient) protein complexes. Proteins in such complexes are usually expressed at very similar rates and should, by analogy to fusions, demonstrate a similar decrease in the evolutionary rate difference.

I do not understand completely the logic behind the quantitative estimation of the expression-based homogenization. To make this estimation the authors consider the correlation between the rates in single domains and the corresponding rate in multi-domain proteins (Figure 6). It seems to me that to estimate the relative contribution one needs also to estimate the variation of the rates for the same orthologous domains. For example, difference in the homogenization obtained by the analysis of human-to-rat versus human-to-mouse orthologs.

**Authors' response: ***The analysis presented here already includes an internal control for rate variation in many evolutionary lineages, namely, those of paralogous domains. Therefore, we believe that the control for variation in orthologous lineages suggested by the reviewer might not be necessary in this case*.

Also the estimation of relative contributions were made by several studies before (I think also by the Koonin et al.) using independent contribution, i.e. partial correlation, analysis. It may be appropriate to comment on these studies.

**Authors' response: ***Somewhat unclear: independent contributions of what? Different evolutionary and functional variables? This is briefly discussed in the text (see Ref. 21)*.

Interestingly, the significantly different homogenization in observed in the case of Arabidopsis compared to the case of human proteins. What can be the reasons for this difference?

**Authors' response: ***Yes, a very curious difference but so far we have no clue as to what it might be owing to*.

### Reviewer's report 3: Claus Wilke (University of Texas-Austin, nominated by Orly Alter) and Alan Drummond (Harvard University, nominated by Joel Bader)

This study quantifies the extent to which protein evolutionary rates are determined by intrinsic structural or functional properties of a protein rather than by external factors, in particular expression level. The study uses a clever method to achieve this goal: it compares the evolutionary rates of individual domains when they are fused into multi-domain proteins to the evolutionary rates of the same domains when they evolve independently. The main result is that both intrinsic and external factors affect domain evolutionary rates to a comparable extent.

This is a wonderful, insightful and important study. Both the method and the results will have an immediate impact on studies of evolutionary rates. We expect that numerous groups (hopefully including the authors) will rapidly extend the present results to other organisms, as the results warrant. In particular, this study emphasizes the role of mutational tolerance in protein domains as a separate issue from the multiplicative role of expression level, a perspective which should be paradigmatic for future investigations.

The authors note that their results fit well with the hypothesis of mistranslation-induced protein misfolding (MIM) which we have recently introduced (Drummond and Wilke, Cell 2008). Under the MIM hypothesis, the selection pressure arises from the fitness cost of misfolded proteins. There is no reason to assume that different domains would have the same propensity to misfold under translation errors, nor that they would impose the same fitness cost when misfolded. Therefore, we expect expression level to homogenize evolutionary rates only to the extent to which two domains have similar misfolding propensities.

The analysis invites a few questions. From the authors' finding that both intrinsic and extrinsic factors contribute roughly equally to the evolutionary rate of domains, it may be tempting to infer that the same conclusion holds for entire genes. Is this inference valid? Perhaps. A way to answer this question in the affirmative would be to show that the evolutionary rate of a multidomain protein is in general well-estimated by the weighted average of the rates for each constituent domain, or that domains cover nearly all of the sequence (in other words, that variation in domain evolutionary rates explains nearly all the variation in gene evolutionary rates). If the evolutionary rates of genes are strongly influenced by features outside of domains, such as the number/length of linker regions, then the inference would be invalid. The authors may at least wish to clarify exactly what their analyses show and what is left to inference.

**Authors' response: ***Our findings for domains would might not quantitatively hold for the whole proteins, in particular, because polypeptide chains outside the domain boundaries have a tendency to fold into disordered or, at least, non-globular structures. However, as long as the regions outside of domains do not dramatically affect the evolutionary rates of the domains themselves, we believe that the present findings can be safely extrapolated to the entire repertoire of the combinations of globular domains*.

Along the same lines, what do we know about the domains being analyzed? Do they appear more often in fast-evolving or slow-evolving genes? Highly expressed or weakly expressed genes? The unstated assumption of the analysis is that the domains show no bias in their origins. Perhaps the assumption could be stated, if not tested, so that we may understand the caveats (if any) in generalizing the present results to whole proteomes.

**Authors' response: ***Again, no assumption is made that the currently detectable structural domains are unbiased "in their origins". For all we know, we might expect the proteins that contain identifiable domains to be biased toward higher "status" (higher expression levels, lower evolutionary rates etc). However, because we compare pairs of domains in multidomain proteins against random pairings of the same domains, there is no need for this domain set to be unbiased relative to other proteins (protein segments) for the conclusions to be valid*.

While the authors have done an admirable job of putting the MIM hypothesis in perspective, at times the language is still even more exuberant than that of the hypothesis' original authors. For example, MIM is referred to as "the dominant factor of evolution" (p.10), whereas in previous works it is referred to as "a dominant constraint on coding-sequence evolution". The difference is nontrivial. The MIM hypothesis was advanced to explain variation in evolutionary rates between coding sequences. The difference in evolutionary rates between an essential protein-coding gene and a random snippet of junk DNA is not mostly due to MIM costs; it is primarily due to selection for a folded, functional polypeptide. The premise of the MIM hypothesis is that, between two coding sequences which are both under pressure to produce functional proteins, that pressure may be more or less constant between them, so that the differences in their evolutionary rates arise mostly from other factors such as MIM.

**Authors' response: ***The readers will, beyond doubt, benefit from these clarifications from the authors of the MIM hypothesis*. *The differences in evolutionary rates between junk DNA (e.g., pseudogenes) and protein-coding genes are indeed substantial, indicating that selection for (any kind of) folded, functional polypeptide is a major factor in evolution compared to which differences between different protein folds might be typically small*.

In their Discussion, the authors nicely divide the mistranslation-induced misfolding (MIM) hypothesis into two parts: a domain's robustness to translation errors to avoid misfolding into a costly molecule, and the cost-multiplier of translation frequency. The description, however, should be modified. For example, "i) Intrinsic misfolding robustness that depends on the structural features of the given domain that are captured by the concept of designability." We believe that designability is a relatively minor contributor to robustness, whereas thermodynamic stability is likely to be a major contributor. Designability, the number of sequences which fold into a particular structure, is a property of a structure, whereas thermodynamic stability is a joint property of the structure and the specific amino-acid sequence. Consider instead: "i) Intrinsic misfolding robustness that depends on the structure and sequence of the given domain. It has been shown."

**Authors' response: ***we agree that stability is likely to be more important than designability; the text was modified essentially as suggested*.

Similarly, factor (i) of the generalized MIM hypothesis states that " [m] is folding of a protein molecule is assumed to incur a specific fitness cost, primarily, through the poisoning effect of misfolded proteins". First, we argue in favor of both direct toxicity (i.e., "poisoning effect") and indirect toxicity (i.e., overwhelming the chaperone machinery so that another insult, such as heat shock, becomes lethal). But more importantly, the nature of the fitness cost is not an assumption of the MIM hypothesis, which only posits that misfolding is costly. The basis of that cost remains an open question, as we (Cell 2008) are at pains to make clear. We do argue (rather than assume!) that the evidence speaks against certain costs, such as loss of function and biosynthetic cost. To avoid confusion, it may be best to say that "misfolding of a protein molecule is assumed to impose a specific fitness cost which may be unrelated to the protein's function, such as through a cytotoxic effect."

**Authors' response: ***The text was modified essentially as suggested*.

We had extensive comments on a previous version of the manuscript; all these comments have been addressed satisfactorily in the revision. We are truly grateful to the authors for their insistence on a careful representation of our previous work, and believe the manuscript is unusually balanced in its presentation.

## Supplementary Material

Additional file 1 Comparison of the domain evolution rate differences in all human proteins and in multidomain proteins (text). Click here for file

Additional file 2 Comparison of the domain evolution rate differences in all *Arabidopsis *proteins and in multidomain proteins (text). Click here for file
